# Supplementary material for: Dimensions of Self-Perceived Functionality in Older Adults Based on the Brazilian National Health Survey
Source: Int J Environ Res Public Health. 2025 Nov 21;22(12):1770. doi: 10.3390/ijerph22121770 (PMC12733159; doi:10.3390/ijerph22121770)

Supplementary Material

**Table S1**. Sociodemographic characteristics of Brazilian older adults included in the 2019 National Health Policy sample.

| Sociodemographic Characteristics of Brazilian Older Adults Included in the Sample | | | | | |
|---|---|---|---|---|---|
| Variable | | Estimates | 95% CI | | Unweighted Count |
| | | | Lower | Upper | |
| SEX | | | | | |
| | M | 43.3% | 42.3% | 44.4% | 10193 |
| | F | 56.7% | 55.6% | 57.7% | 12535 |
| | Total | 100.0% | 100.0% | 100.0% | 22728 |
| Federative Unit | | | | | |
| | North | 6.1% | 5.8% | 6.4% | 3487 |
| | Northeast | 25.4% | 24.6% | 26.2% | 7736 |
| | Southeast | 46.4% | 45.4% | 47.5% | 5825 |
| | South | 15.7% | 15.1% | 16.4% | 3307 |
| | Central-West | 6.4% | 6.0% | 6.7% | 2373 |
| | Total | 100.0% | 100.0% | 100.0% | 22728 |
| Weekly Exercise Time (Accumalated) | | | | | |
| | Not active | 38.9% | 36.9% | 40.9% | 2558 |
| | Active | 61.1% | 59.1% | 63.1% | 4057 |
| | Total | 100.0% | 100.0% | 100.0% | 6615 |
| Resident's Age on Reference Date | Mean | 69.86 | 69.68 | 70.03 | 22728 |
| Number of Chronic Noncommunicable Diseases (CNCDs) | Mean | 2.2375 | 2.1960 | 2.2789 | 21725 |
| Race or color | | | | | |
| | White | 50.5% | 49.4% | 51.7% | 9901 |
| | Black | 10.3% | 9.6% | 11.0% | 2455 |
| | Asian | 1.3% | 1.0% | 1.6% | 204 |
| | Brown | 37.4% | 36.3% | 38.5% | 10001 |
| | Indigenous | 0.5% | 0.4% | 0.7% | 165 |
| | Without declaration | 0.0% | 0.0% | 0.0% | 2 |
| | Total | 100.0% | 100.0% | 100.0% | 22728 |
| Spouse/Partner Lives in Household | Yes | 56.3% | 55.2% | 57.3% | 10725 |
| | No | 43.7% | 42.7% | 44.8% | 12003 |
| | Total | 100.0% | 100.0% | 100.0% | 22728 |
| Literate (Can Read and Write) | Yes | 81.4% | 80.5% | 82.2% | 17345 |
| | No | 18.6% | 17.8% | 19.5% | 5383 |
| | Total | 100.0% | 100.0% | 100.0% | 22728 |
| Highest Level of Education Attended | Daycare | 0.1% | 0.0% | 0.1% | 9 |
| | Preschool | 0.4% | 0.3% | 0.5% | 122 |
| | Literacy class | 4.2% | 3.7% | 4.6% | 1114 |
| | Youth and Adult Literacy | 1.3% | 1.1% | 1.5% | 283 |
| | Former Primary Education | 40.4% | 39.1% | 41.6% | 7271 |

| | | | | | |
|---|---|---|---|---|---|
| | Former Middle School | 8.5% | 7.7% | 9.3% | 1422 |
| | Regular Elementary Education (or 1st Grade) | 9.1% | 8.5% | 9.8% | 2074 |
| | Youth and Adult Education (EJA) - Elementary Level | 1.7% | 1.4% | 2.0% | 343 |
| | Former Scientific/Classic Education (High School - 2nd Cycle) | 6.0% | 5.5% | 6.6% | 1054 |
| | Regular High School (or 2nd Grade) | 12.6% | 11.8% | 13.4% | 2325 |
| | Youth and Adult Education (EJA) - High School Level | 1.1% | 0.8% | 1.4% | 199 |
| | Higher Education - Undergraduate Degree | 11.2% | 10.4% | 12.1% | 1954 |
| | Specialization (Minimum 360 Hours) | 2.7% | 2.3% | 3.1% | 535 |
| | Master's Degree | 0.6% | 0.5% | 0.8% | 128 |
| | Doctorate | 0.3% | 0.2% | 0.4% | 62 |
| | Total | 100.0% | 100.0% | 100.0% | 18895 |

Component Statistics

**Table S2**. Summary of Principal Component Analysis (PCA): Eigenvalues, Variances, and Cumulative Variances.

| **Component** | **Eigenvalue** | **% of Total Variance** | **% Cumulative Variance** |
|---|---|---|---|
| ADLs | 5.07 | 42.3 | 42.3 |
| IADLs | 4.15 | 34.6 | **76.9** |

The bolded value highlights that the first two components together capture more than 70% of the cumulative variance in the Principal Component Analysis (PCA).

**Table S3.**
Description of the components, eigenvalues, variances, and cumulative variances of the PCA for ADLs.

| **Component** | **Eigenvalue** | **Variance %** | **Cumulative Variance %** |
|---|---|---|---|
| 1 | 5.3475 | 76.39 | **76.4** |
| 2 | 0.7033 | 10.05 | 86.4 |
| 3 | 0.3471 | 4.96 | 91.4 |
| 4 | 0.2054 | 2.93 | 94.3 |
| 5 | 0.1637 | 2.34 | 96.7 |
| 6 | 0.1461 | 2.09 | 98.8 |

| Component | Eigenvalue | Variance % | Cumulative Variance % |
|---|---|---|---|
| 7 | 0.0869 | 1.24 | 100.0 |

The bold value highlights that the first component captures the highest cumulative variance in the Principal Component Analysis (PCA), exceeding 70%.

**Table S4.**
Description of the components, eigenvalues, variances, and cumulative variances of the PCA for IADLs.

| Componente | Eigenvalue | Variance % | Cumulative Variance % |
|---|---|---|---|
| 1 | 3.802 | 76.03 | **76.0** |
| 2 | 0.536 | 10.71 | 86.7 |
| 3 | 0.344 | 6.87 | 93.6 |
| 4 | 0.204 | 4.08 | 97.7 |
| 5 | 0.115 | 2.31 | 100.0 |

The bold value highlights that the first component captures the highest cumulative variance in the Principal Component Analysis (PCA), exceeding 70%.

**Figure S1**
Description of the Scree plot of the components evaluated by PCA.

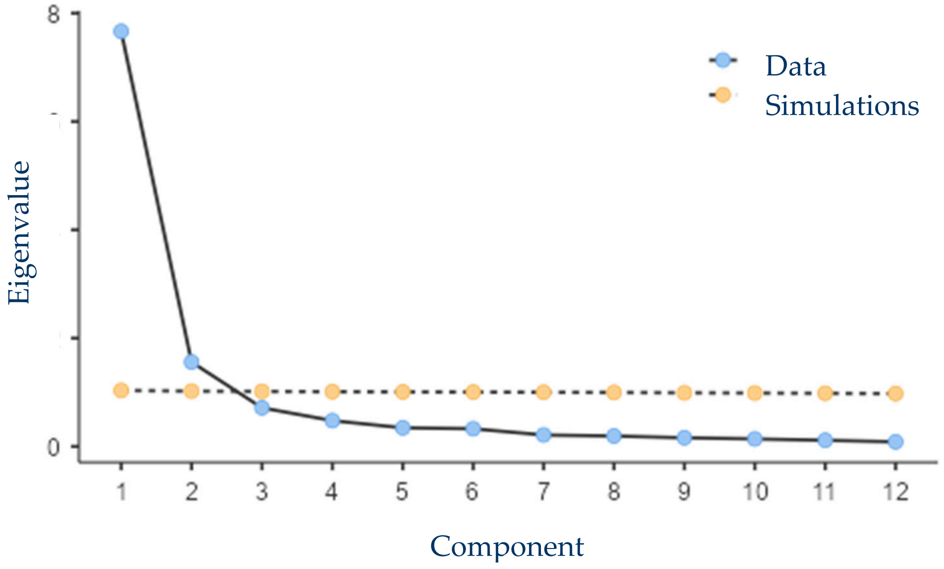

Supplement: Supplementary file 1 [file ijerph-22-01770-s001.zip › ijerph-3597294-supplementary.pdf]
